# The Effects of Transcutaneous Spinal Direct Current Stimulation on Neuropathic Pain in Multiple Sclerosis: Clinical and Neurophysiological Assessment

**DOI:** 10.3389/fnhum.2019.00031

**Published:** 2019-02-12

**Authors:** Eliana Berra, Roberto Bergamaschi, Roberto De Icco, Carlotta Dagna, Armando Perrotta, Marco Rovaris, Maria Grazia Grasso, Maria G. Anastasio, Giovanna Pinardi, Federico Martello, Stefano Tamburin, Giorgio Sandrini, Cristina Tassorelli

**Affiliations:** ^1^Neurorehabilitation Unit, Department of Neurology, IRCCS C. Mondino Foundation, Pavia, Italy; ^2^Multiple Sclerosis Center, IRCCS C. Mondino Foundation, Pavia, Italy; ^3^Department of Brain and Behavioral Sciences, University of Pavia, Pavia, Italy; ^4^Department of Clinical-Surgical, Diagnostic and Pediatric Sciences, University of Pavia, Pavia, Italy; ^5^IRCCS Neuromed, Pozzilli, Italy; ^6^Neurorehabilitation Unit and Multiple Sclerosis Center, IRCCS Santa Maria Nascente, Don Carlo Gnocchi Foundation, Milan, Italy; ^7^Multiple Sclerosis Unit, Rehabilitation Hospital Santa Lucia Foundation, Rome, Italy; ^8^Department of Neurosciences, Biomedicine and Movement Sciences, University of Verona, Verona, Italy

**Keywords:** neuropathic pain, transcutaneous spinal direct current stimulation (ts-DCS), non-invasive, nociceptive withdrawal reflex, neuromodulation, multiple sclerosis

## Abstract

**Background**: Central neuropathic pain represents one of the most common symptoms in multiple sclerosis (MS) and it seriously affects quality of life. Spinal mechanisms may contribute to the pathogenesis of neuropathic pain in MS. Converging evidence from animal models and neurophysiological and clinical studies in humans suggests a potential effect of transcranial direct current stimulation (tc-DCS) on neuropathic pain. Spinal application of DCS, i.e., transcutaneous spinal DCS (ts-DCS), may modulate nociception through inhibition of spinal reflexes. Therefore, ts-DCS could represents an effective, safe and well-tolerated treatment for neuropathic pain in MS, a largely unexplored topic. This study is a pilot randomized double-blind sham-controlled trial to evaluate the efficacy of ts-DCS on central neuropathic pain in MS patients.

**Methods**: Thirty-three MS patients with central neuropathic pain were enrolled and randomly assigned to two groups in a double-blind sham-controlled design: anodal ts-DCS group (*n* = 19, 10 daily 20-min sessions, 2 mA) or sham ts-DCS group (*n* = 14, 10 daily 20-min sessions, 0 mA). The following clinical outcomes were evaluated before ts-DCS treatment (T0), after 10 days of treatment (T1) and 1 month after the end of treatment (T2): neuropathic pain symptoms inventory (NPSI), Ashworth Scale (AS) for spasticity and Fatigue Severity Scale (FSS). A subgroup of patients treated with anodal ts-DCS (*n* = 12) and sham ts-DCS (*n* = 11) also underwent a parallel neurophysiological study of the nociceptive withdrawal reflex (NWR) and the NWR temporal summation threshold (TST), two objective markers of pain processing at spinal level.

**Results**: Anodal ts-DCS group showed a significant improvement in NPSI at T1, which persisted at T2, while we did not detect any significant change in AS and FSS. Sham ts-DCS group did not show any significant change in clinical scales. We observed a non-significant trend towards an inhibition of NWR responses in the anodal ts-DCS group at T1 and T2 when compared to baseline.

**Conclusions**: Anodal ts-DCS seems to have an early and persisting (i.e., 1 month after treatment) clinical efficacy on central neuropathic pain in MS patients, probably through modulation of spinal nociception.

**Clinical Trial Registration:** www.ClinicalTrials.gov, identifier #NCT02331654.

## Introduction

Pain represents one of the most disabling symptoms of multiple sclerosis (MS), in that it adversely affects most aspects of health-related quality of life and is often neglected and undertreated. The overall prevalence of pain syndromes in MS patients is 63% (Foley et al., 2013), with a higher risk associated with older age, longer disease duration, and greater disease severity (Solaro et al., 2004). A recent multicenter cross-sectional study on 1,249 MS patients reported a 33.8% overall frequency of pain (Solaro et al., 2018). Central neuropathic pain, defined as a pain that is directly caused by a lesion or disease involving the somatosensory system (Treede et al., 2008), is the main type category of MS-related pain and it can be distinguished in three main forms: central neuropathic (“dysesthetic”) extremity pain, trigeminal neuralgia and Lhermitte’s sign (O’Connor et al., 2008).

Central neuropathic extremity pain is described as a continuous burning, tingling or aching pain, unilateral or bilateral in distribution, affecting legs and feet, even in the early stages of the disease. This type of pain is thought to be caused by lesions in brain and spinal cord nociceptive pathways leading to a dysregulation in inhibitory and/or excitatory pain mechanisms, including GABA-ergic interneurons and NMDA receptors (Olechowski et al., 2013). This type of MS-related pain has a prevalence ranging from 16 to 26% (Foley et al., 2013; Solaro et al., 2018) and its treatment remains a significant challenge as available therapies are scarcely effective or poorly tolerated (O’Connor et al., 2008; Paolucci et al., 2016).

Patients with MS may also report nociceptive types of pain that include painful tonic spasms related to corticospinal tract involvement, musculoskeletal and back pain (Solaro et al., 2004, 2018).

Transcranial direct current stimulation (tc-DCS) represents a non-invasive, safe and well tolerated method for selectively modulating cortical excitability in a polarity-dependent way. Tc-DCS was documented to have an impact on a range of motor (Nitsche and Paulus, 2001; Nitsche et al., 2005), somatosensory (Rogalewski et al., 2004; Grundmann et al., 2011), visual (Antal et al., 2006), affective and cognitive functions (Kincses et al., 2004; Boggio et al., 2008) and it has received increased attention regarding potential therapeutic applications in the fields of neurology and psychiatry. Up to now, the therapeutic effects of tc-DCS are considered promising for fibromyalgia and major depressive disorders and emerging evidence has underlined its great potential efficacy in pain conditions (Antal et al., 2008; Boggio et al., 2008; Csifcsak et al., 2009; Hansen et al., 2011). However, convincing evidence on the efficacy of tc-DCS of the motor cortex in chronic pain conditions needs is still missing (O’Connell et al., 2011). Anodal tc-DCS of the left motor cortex, or contralateral to the pain side, with right orbitofrontal cathode, has indeed received a level C recommendation (i.e., possible efficacy) in chronic lower limb neuropathic pain secondary to spinal cord lesion (Lefaucheur et al., 2017).

The spinal application of DCS, defined as transcutaneous spinal direct current stimulation (ts-DCS), may represent a non-invasive, safe, non-pharmacological and potentially self-administered approach to those conditions where pain is generated or becomes chronic through changes in spinal interneurons. In humans, anodal ts-DCS has been proven to inhibit nociceptive specific responses, such as the nociceptive withdrawal reflex (NWR; Cogiamanian et al., 2011) and the NWR temporal summation threshold (TST; Perrotta et al., 2016). The hypothesized mechanism involved in the modulation of the NWR responses could be direct or supraspinal-mediated change in excitability of spinal sensory neurons, including the wide dynamic range (WDR) neurons mediating short-term NMDA-mediated plasticity, which is involved in the spinal cord pain processing as well as in and the genesis and maintenance of chronic pain (Perrotta et al., 2016).

Based on these pieces of evidence, ts-DCS might be a valuable therapeutic approach in subjects with MS and diagnosed with central neuropathic extremity pain. However, the efficacy of ts-DCS in this type of pain has been seldom investigated in humans.

### Aim of the Study

The aim of this pilot multi-center randomized controlled trial (RCT) is to evaluate the short and middle-term effect of treatment with anodal ts-DCS on central neuropathic extremity pain in MS, using clinical scales and neurophysiological measures of spinal nociception. We focused on central neuropathic extremity pain, because it is by far the most common type of neuropathic pain in MS (Foley et al., 2013; Solaro et al., 2018). The primary outcome measure was neuropathic pain severity, and the secondary endpoints were the neurophysiological measures derived from the NWR. Other secondary measures included spasticity and fatigue.

## Materials and Methods

This was a multicentric study carried out in accordance with the Guidelines for Good Clinical Practice. The protocol was approved by the Ethics Committee of the Coordinating Center (Mondino Foundation, Approval number: 2786/13) and subsequently approved by the Local Institutional Ethical Committees of the participating centers. Before enrollment, all subjects gave their written informed consent in accordance with the Declaration of Helsinki.

### Subjects

Thirty-three subjects (25 female and 8 male) with a definite diagnosis of MS according to McDonald criteria (Polman et al., 2011), and suffering from neuropathic pain of the lower limbs, were recruited in a double-blind, placebo-controlled, multicenter study design (NCT02331654).

Neuropathic pain was diagnosed according to the grading system (Treede et al., 2008) and the Douleur Neuropathique Questionnaire 4 (DN4; Bouhassira et al., 2005). Patients were diagnosed as suffering from definite central neuropathic extremity pain when other likely causes of pain were excluded, pain had a plausible neuroanatomical distribution confirmed by clinical findings, the DN4 score was ≥4 and neuroimaging showed a demyelinating lesion consistent with the pain distribution (Treede et al., 2008; Solaro et al., 2018).

Patients were recruited at the IRCCS C. Mondino Foundation in Pavia, Santa Lucia Foundation in Rome, IRCCS “Neuromed” Institute in Pozzilli and Don Gnocchi Foundation in Milan, Italy.

Relapsing-remitting (RR), secondary-progressive (SP) and primary-progressive (PP) MS patients were enrolled in a follow-up procedure that included a general and neurological evaluation scored according to the Expanded Disability Status Scale of Kurtzke and its functional systems (Kurtzke, 1983). RR patients were evaluated in a stationary phase of the disease, at least 2 months after the last clinical relapse and at least 1 month after the end of a steroidal treatment.

For all participants, exclusion criteria were: (a) other neurological disorders, including primary or secondary headaches; (b) clinical or family history of neurological disorders; (c) any systemic or psychiatric disorder; (d) Beck Depression Inventory (BDI) scale score >9; (e) cognitive impairment (Mini Mental State Examination < = 24); (f) use of analgesics or steroids in the previous 24 h; (g) clinical or instrumental (including MRI) evidence of any central or peripheral disease/lesion potentially causing sensory impairment, including spinal lesions at lumbar level; (h) fibromyalgia; (i) complex regional pain syndrome; (j) chronic low back pain and other pain conditions not related to MS; and (k) changes in the schedule or dose of Disease Modifying Drugs (DMDs) for MS, antidepressants, antiepileptic drugs, tetrahydrocannabinol/cannabidiol or any other drug that may have a definite or potential effect on pain in the previous 3 months. Patients were excluded from the study if:

- any change in the schedule or dose of drugs listed at point (l) above became necessary at any time during the observation period.
- they had taken analgesics or steroids in the 24 h before the clinical and neurophysiological evaluations.

Patients were randomly assigned to two groups: 19 patients were assigned to the anodal ts-DCS treatment group and 14 patients to the sham ts-DCS group. A subgroup of 12 patients treated with anodal ts-DCS and 11 patients treated with sham ts-DCS also underwent the neurophysiological evaluation of the NWR (see below).

### Transcutaneous Spinal Direct Current Stimulation (ts-DCS)

Anodal and sham ts-DCS was delivered by a constant direct current electrical stimulator (HDCstim, Newronika s.r.l., Milan, Italy) connected to a pair of electrodes: the anode was placed on the thoracic spinal cord (over the spinal process of the tenth thoracic vertebra) and the cathode (reference) on the right shoulder in the suprascapular region. Stimulating electrodes consisted in 1-mm thick, rectangular (7 × 5 cm), rubber membranes, enveloped in a saline-soaked sponge. Conducting surface was 35 cm^2^ for both active and reference electrode. Electrodes were fixed inside by elastic customized stripes.

For the real anodal ts-DCS group, we delivered a 2 mA constant direct current for 20 min in each session with a density of 0.071 mA/cm^2^ and delivered a total charge of 63.9 mC/cm^2^. These parameters are far below both the threshold for tissue damage and the conscious sensory threshold, apart from transient, and short-lasting tingling sensation below the electrodes at the start of the stimulation.

For the sham ts-DCS group, electrodes were placed in the same spots than real anodal stimulation, but the stimulator was programmed to automatically turn to 0 mA after 10 s.

We based the choice of the stimulation site and the related stimulation parameters on the results of our previous study in healthy subjects in which we demonstrated the effectiveness of the tsDCS in modulating the TST of the NWR (Perrotta et al., 2016).

The treatment protocol consisted of 10 daily 20-min sessions of active or sham ts-DCS delivered over a 2-week period (from Monday to Friday) and a follow-up period of 4 weeks. Patients and assessing physicians were blind to group allocation; stimulator programming and electrodes applications were made by physicians not involved in the enrollment, clinical and neurophysiological evaluation, and data analysis.

### Clinical Assessments

Patients were evaluated with clinical scales at baseline (T0), at the end of the 2-week treatment period (T1) and at 1 month from the end of treatment (T2). Characteristics and intensity of pain symptoms were collected at each stage with the Validated Italian Version of Neuropathic Pain Symptoms Inventory (NPSI; Padua et al., 2009). Spasticity of lower legs, if present, was assessed with the Ashworth Scale (AS; Pandyan et al., 1999). The presence and severity of fatigue was assessed by means of the Fatigue Severity Scale (FSS; Krupp et al., 1989).

### Neurophysiological Testing: Sensory Threshold, Nociceptive Withdrawal Reflex Threshold and Temporal Summation Threshold

In parallel with clinical evaluation, the previously described subgroup of MS patients underwent a neurophysiological testing that included sensory threshold (Sth), NWR threshold (Rth), area under curve response (Area) and NWR TST and was administered at T0, T1 and T2.

The NWR from the lower limbs was investigated according to a previously described and validated method (Sandrini et al., 2005). Subjects were seated in a comfortable armchair in a quiet room at constant temperature. Their lower limbs were positioned to ensure complete muscle relaxation with knee flexed at 130° and ankle at 90°.

The sural nerve was stimulated percutaneously *via* a pair of standard Ag/AgCl surface electrodes applied to degreased skin behind the right lateral malleolus. The stimulus consisted of 20 ms current pulse train of five individual 1 ms rectangular pulses delivered at 200 Hz (equal to an inter-stimulus interval of 4 ms). Electromyographic reflex responses were recorded from the capitis brevis of the biceps femoris muscle *via* a standard pair of Ag/AgCl surface electrodes. The analysis time was 300 ms, with the sensitivity set at 100 mV, the filter bandpass setting was between 3 Hz and 3 kHz (CED Powerlab interface 1401, Cambridge Electronic Design, UK; electronic amplifier BM623, Biomedica Mangoni, Italy; electric simulator DS7A, Digitimer, UK).

In order to evaluate the efficiency of the sensory pathways, the Sth was determined at the start of each experimental session. The previously described pulse train, increased or decreased in 0.1 mA steps were delivered at unpredictable intervals of ±10 s. Subjects were asked to indicate verbally the stimulation levels at which they became aware of sensory sensations. The sensory threshold was defined as the lowest stimulation intensity generating a stable sensory sensation.

The staircase method was used to evaluate the Rth, defined as the lowest stimulation intensity generating a clearly detectable nociceptive reflex response exceeding 20 μV for 10 ms or more in the time interval 80–130 ms over five pulse train randomly delivered every 20–40 s and hence the related pain perception. The intensity of stimulation was then fixed at 1.2 Th and randomly delivered every 5–20 s to avoid habituation phenomena. Each response was full-wave rectified and integrated between set points from 80 to 130 ms after the start of the test stimulus. Five reflex responses were recorded and the mean NWR Area was computed by means of a computerized method.

To evaluate the NWR TST, the previously described stimulation/recording setting was used. The pulse train of five individual 1-ms pulses delivered at 200 Hz was repeated five times at a frequency of 2 Hz. The stimulus intensity was increased (in 1 mA steps) from 2 mA until detection of temporal summation of the NWR. The NWR TST was defined as the lowest stimulation intensity generating a stable NWR response at the fourth and fifth pulse train of the five-train series and exceeding 20 μV in the time interval between 80 and 130 ms for a period of more than 10 ms. A threshold was accepted when three consecutive recordings resulted in the same threshold. Subjects rated the subjective pain sensation for each stimulus on a 0–11 numerical rating scale, (NRS, graded from 0 = no pain to 10 = unbearable pain, with pain sensation anchored to 5). The mean psychophysical pain sensation at Th (NRS at Th) and at first (NRS1) and fifth (NRS5) stimulus of the five delivered to evoke the TST was calculated.

### Data Analysis

The Statistical Package for the Social Sciences (SPSS) for Windows, version 21.0, was used for the calculation. For each variable we evaluated skewness and kurtosis to assess normality. Moreover, the data were plotted using a “Q-Q plot” that confirmed normal distribution of all tested variables. For qualitative variables we used cross-tabs analysis, performing statistical significance with chi-square or Fisher exact test. Quantitative variables are presented as mean values ± standard deviation. To assess the intragroup acute effect, we used a Student’s *t*-test for related samples or, in presence of multiple time measurements, a repeated measures analyses of variance (ANOVA) and *post hoc* with Bonferroni’s correction. To assess differences between groups at each time point we used a Student’s *t*-test for unpaired samples. The level of significance (α) was set for convention as *p* = 0.05 for all the tests, and always corrected where necessary.

## Results

Demographic features and baseline parameters of participating groups are reported in Table 1. No statistically significant differences in clinical parameters were detected between groups at T0.

**Table 1:** Demographic and clinical characteristics of the active and sham ts-DCS groups.

Demographic and clinical characteristics	Active ts-DCS (*n* = 19)	Sham ts-DCS (*n* = 14)	*p* value
Age (years)	57.6 ± 9.1	54.0 ± 7.79	n.s.
Sex			n.s.
Men	4 (21.1%)	4 (28.6%)	
Women	15 (78.9%)	10 (71.4%)	
Multiple sclerosis type			n.s.
Relapsing remitting	1 (5.3%)	3 (21.4%)	
Secondary progressive	14 (73.7%)	10 (71.4%)	
Primary progressive	4 (21.1%)	1 (7.1%)	
Disease duration (years)	19.7 ± 8.8	15.9 ± 7.5	
Relapsing remitting	16.0 ± 0.0	12.7 ± 5.7	
Secondary progressive	24.5 ± 7.1	21.0 ± 9.3	n.s.
Primary progressive	18.6 ± 10.5	14.0 ± 0.0	
EDSS score	5.9 ± 1.3	5.9 ± 1.2	n.s.
Pharmacological Treatments			
Disease Modifying Drugs (DMDs)	7 (36.8%)	5 (35.7%)	
Tetrahydrocannabinol/Cannabidiol	3 (15.7%)	3 (21.4%)	n.s.
Other drugs for neurophatic pain	8 (42.1%)	7 (50.0%)	

*Data are reported as mean ± SD or N (%). ts-DCS, transcutaneous spinal direct current stimulation; EDSS, Kurtzke Expanded Disability Status Scale; n.s, not significant (*p* value > 0.05)*.

### Clinical Evaluation

**Figure 1:**
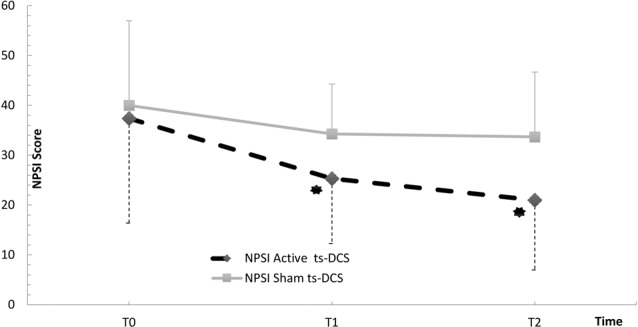
NPSI Score in the active and sham ts-DCS Groups. Data are reported as mean ± SD. ts-DCS, transcutaneous spinal direct current stimulation; NPSI, Neuropathic Pain Symptoms Inventory. *Significant active ts-DCS vs. sham ts-DCS comparison (*p* value < 0.05).

In the active ts-DCS group, we found a significant reduction in NPSI mean values (*F*_(2,17)_ = 5.175; *p* = 0.013) at both T1 (Bonferroni’s corrected paired *t*-test: *p* = 0.023) and T2 (*p* = 0.008) when compared to T0 (Table 2; Figure 1). No significant differences were seen at any time point in AS and FSS (Table 2).

**Table 2:** Clinical outcomes in the active and sham ts-DCS groups.

Outcome	Active ts-DCS (*n* = 19)						Sham ts-DCS (*n* = 14)					
					*Post hoc* (*p* value)						*Post hoc* (*p* value)	
	T0	T1	T2	Time	T1 vs. T0	T2 vs. T0	T0	T1	T2	Time	T1 vs. T0	T2 vs. T0
NPSI	37.4 ± 21.4	25.3 ± 13.8	21.0 ± 14.4	*F*_(2,17)_ 5.175; *p* = 0.013	0.023	0.008	40.0 ± 17.9	34.3 ± 10.2	33.7 ± 13.2	*F*_(2,12)_ 1.434; *p* = 0.277	n.s.	n.s.
FSS	41.8 ± 13.4	41.1 ± 14.1	37.6 ± 11.5	*F*_(2,17)_ 1.177; *p* = 0.331	n.s.	n.s.	49.0 ± 12.1	47.2 ± 11.0	48.5 ± 13.0	*F*_(2,12)_ 0.330; *p* = 0.725	n.s.	n.s.
AS	1.75 ± 0.8	1.8 ± 0.8	1.8 ± 1.1	*F*_(2,17)_ 0.643; *p* = 0.537	n.s.	n.s.	1.2 ± 0.9	1.5 ± 1.2	1.2 ± 0.6	*F*_(2,12)_ 1.200; *p* = 0.335	n.s.	n.s.

*Data are reported as mean ± SD. ts-DCS, transcutaneous spinal direct current stimulation; NPSI, Neuropathic Pain Symptoms Inventory; FSS, Fatigue Severity Scale; AS, Ashworth Scale; n.s., not significant (*p* value > 0.05)*.

In the sham ts-DCS group, we did not detect any significant change in any of the clinical variables considered (i.e., NPSI, AS, FSS; Table 2).

The intergroup analysis showed a significant improvement in the NPSI score in the active ts-DCS group compared to the sham ts-DCS group at both T1 (*F*_(1,31)_ 4.194; *p* = 0.049) and T2 (*F*_(1,31)_ 6.637; *p* = 0.015; Figure 1). AS and FSS scores did not differ between groups at any time point.

### Neurophysiological Evaluation

We observed a tendency towards an increase in the neurophysiological parameters (Rth, NWR TST) in the active ts-DCS group at T1 when compared to T0, suggesting a reduced pain processing. However, no significant difference was found in either active or sham ts-DCS groups when comparing the neurophysiological and related psychophysical parameters recorded at T1 or T2 with T0 values (Table 3). Similarly, intergroup analysis did not show any significant change between active and sham ts-DCS groups in Sth, Rth and TST at T0, T1 and T2.

**Table 3:** Neurophysiological features in the active and sham ts-DCS groups.

Outcome	Active ts-DCS (*n* = 12)						Sham ts-DCS (*n* = 11)					
					*Post hoc* (*p* value)						*Post hoc* (*p* value)	
	T0	T1	T2	Time	T1 vs. T0	T2 vs. T0	T0	T1	T2	Time	T1 vs. T0	T2 vs. T0
Sth (mA)	0.76 ± 0.2	0.88 ± 0.6	0.98 ± 0.6	*F*_(2,12)_ 0.445; *p* = 0.677	n.s.	n.s.	0.70 ± 0.3	0.72 ± 0.3	0.73 ± 0.3	*F*_(2,9)_ 0.199; *p* = 0.823	n.s.	n.s.
Rth (mA)	17.54 ± 8.5	20.57 ± 13.2	16.95 ± 9.1	*F*_(2,12)_ 2.156; *p* = 0.167	n.s.	n.s.	16.92 ± 8.9	16.06 ± 9.2	16.62 ± 9.2	*F*_(2,9)_ 0.301; *p* = 0.747	n.s.	n.s.
Area	2332.7 ± 1546.7	1475.1 ± 961.0	1581.3 ± 1469.6	*F*_(2,10)_ 2.153; *p* = 0.167	n.s.	n.s.	1534.9 ± 1210.5	1072.6 ± 627.9	1573.6 ± 786.6	*F*_(2,8)_ 2.178; *p* = 0.176	n.s.	n.s.
TST (mA)	8.42 ± 3.9	10.09 ± 5.7	7.79 ± 4.6	*F*_(2,3)_ 0.511; *p* = 0.644	n.s.	n.s.	8.26 ± 3.7	7.69 ± 4.4	7.62 ± 3.6	*F*_(2,7)_ 0.247; *p* = 0.788	n.s.	n.s.

*Data are reported as mean ± SD. Sth, Sensory Threshold; Rth, nociceptive withdrawal reflex threshold; TST, temporal summation threshold; n.s., not significant (*p* value > 0.05)*.

## Discussion

The main result of this study is that a 2-week period of daily anodal ts-DCS at the thoracic level induced a significant and persisting improvement in pain severity in patients with MS and central neuropathic extremity pain. Indeed, the observed improvement in pain symptoms persisted for further 4 weeks after the end of the stimulation period. On the contrary, the sham stimulation did not influence clinical parameters, (i.e., pain, spasticity and fatigue) in the control group. Neurophysiological parameters, including Rth and TST of the NWR, showed a parallel pattern to the clinical findings when considering the T0 and T1 time point, although the neurophysiological changes did not reach a statistically significant level. Taken as a whole, these findings are in line with previous reports of inhibition of pain reflex responses after anodal ts-DCS in healthy subjects (Cogiamanian et al., 2011; Perrotta et al., 2016), and of clinical improvement of central neuropathic pain after tc-DCS in subjects with MS (Mori et al., 2010).

Based on the present data, which showed ts-DCS to be effective on MS-related neuropathic pain, and to partially inhibit nociceptive reflex responses driven by WDR neurons, we hypothesize that ts-DCS may modulate segmental or intersegmental excitability in spinal nociceptive neurons, which are likely to be the WDR neurons, as observed in healthy subjects (Cogiamanian et al., 2011; Perrotta et al., 2016).

Spinal and trigeminal WDR neurons can plastically change their excitability in a graded manner, as function of the frequency and the intensity of the stimulation, in a phenomenon known as wind-up (Liebetanz et al., 2002; Mendell and Wall, 1965). This property of the WDR neurons induces a shift from tactile to painful sensory processing, and it is considered to be pivotal in physiological nociception for the discriminative analysis of pain sensation (Herrero et al., 2000; Perrotta et al., 2017), and in the pathogenesis of chronic pain for the induction and maintenance of central sensitization (Li et al., 1999; Eide, 2000; Perrotta et al., 2010). Thus, we speculate the parallel inhibition of NWR TST in healthy subjects (Cogiamanian et al., 2011; Perrotta et al., 2016) and the improvement of MS-related neuropathic pain after ts-DCS to be a consequence of long-lasting WDR neurons excitability depression. In addition, the excitability of the WDR neurons is strictly associated to the activity of the NMDA receptors (Dickenson and Sullivan, 1987; Guirimand et al., 2000), which are likewise considered to be involved in the pathogenesis of neuropathic pain in MS (Olechowski et al., 2013). Based on the observation that cortical excitation to both anodal and cathodal tc-DCS is prevented by NMDA blockade (Liebetanz et al., 2002; Nitsche et al., 2003), we may hypothesize the observed modulation of pain processing to ts-DCS to be similarly caused by a change in NMDA receptor activity at the spinal level. The lack of inhibitory effect of thoracic ts-DCS on both evoked responses at cervical level (Cogiamanian et al., 2008) and H-reflex excitability (Cogiamanian et al., 2011), makes the involvement of supraspinal antinociceptive descending projections and/or motoneuron inhibition quite unlikely mechanisms of action.

However, a series of issues that have emerged from our data need to be carefully considered and discussed. We found a non-significant trend toward a TST depression in the ts-DCS group. This finding could be related either to the small sample size of the group undergoing neurophysiological evaluation, or to specific features of our MS patients. MS is a complex disease and progressive forms of MS are associated with neurodegeneration (Baecher-Allan et al., 2018). Most patients in our study were affected by SP MS, had a longer disease duration and spinal lesions. We speculate that these features, which are frequently associated with neurodegeneration and largely abnormal or unobtainable evoked potentials, may have interfered with NWR modulation by ts-DCS. However, though a comparison of the present NWR findings to the few previously published ones is difficult, due to sample sizes and methodological issues, our findings are in line with the observed inhibition of the NWR responses in MS patients treated with drugs for pain and spasticity (e.g., topiramate, gabapentin, tetrahydrocannabinol/cannabidiol; Conte et al., 2009; Foley et al., 2013). Some of our patients were on tetrahydrocannabinol/cannabidiol, but the stability of the dose (at least 3 months before enrolment and across the entire study) suggest that intake of this drug is unlikely to have influenced our clinical and neurophysiological findings.

We could not document any significant change in spasticity and fatigue scores to ts-DCS. This finding is in keeping with previous controversial data on the effect of DCS on these two outcomes (Palm et al., 2014; Iodice et al., 2017).

Indeed, previous reports yielded discordant data on the effect of neuromodulation technique on spasticity (Khan et al., 2017). A Cochrane review underlined a low-quality evidence for the use of TMS in MS-related spasticity (Amatya et al., 2013), and controversial data are reported on tc-DCS (Khan et al., 2017). Few data about the effect of ts-DCS on muscle tone are available (Ahmed, 2014), and none is specific for MS patients.

In our opinion, the lack of effectiveness of ts-DCS on motoneuron excitability (Cogiamanian et al., 2011), the complex mechanisms involved in MS spasticity, and the specific features of our MS patients (i.e., prevalence of SP forms and spinal lesions) could explain the lack of effect of ts-DCS on spasticity.

Fatigue represents a frequent and invalidating symptom in MS. MS fatigue is still considered the result of a multifactorial and complex constellation, and is commonly classified into “primary” fatigue, i.e., related to the pathological MS changes, and “secondary” fatigue, which is attributed to mimicking symptoms, comorbid sleep and mood disorders, medications side effects, spasticity, and pain (Chalah et al., 2015). Primary fatigue is linked to brain atrophy and supraspinal lesions that alter cortico-striato-thalamo-cortical connectivity. Evidence in MS patients suggested that tc-DCS of the motor (Ferrucci et al., 2014) or the sensory cortex (Tecchio et al., 2014) may improve fatigue, especially the primary one or that related with mood disorders. Recent studies suggest that ts-DCS could improve fatigue resistance and enhance some physical performances (e.g., vertical jump) in healthy subjects (Berry et al., 2017). Possible reasons for the absence of significant effect of ts-DCS on fatigue in our study may include the clinical features of our patients (i.e., long duration of disease, presence of pain, spasticity, and hypasthenia), which might be mainly related to secondary fatigue, and the spinal site of stimulation. In contrast to pain, only supraspinal mechanisms seem to be altered in MS-related fatigue (Chalah et al., 2015), and this point could explain the higher efficacy of tc-DCS than ts-DCS on this symptom.

### Limitations

This study was conducted on a small number of patients with a long disease duration, who may not be representative of the full population of MS patients and prevented us from stratifying our population by disease form (i.e., RR, SP, or PR) or lesion load site (i.e., mainly cortical or spinal). Further studies are needed to evaluate the effect of ts-DCS on different types of MS-related pain, either neuropathic or nociceptive. Moreover, studies that stratify patients according to disease type and the presence or spinal lesions could better clarify mechanism of ts-DCS in MS.

## Conclusion

In conclusion, our results suggest that ts-DCS may improve central neuropathic pain in MS patients for up to 4 weeks. Spinal DCS could be a promising, non-invasive, well-tolerated and potentially self-administered treatment for neuropathic pain in MS patients. This type of DCS may play a role as an add-on treatment in drug-resistant pain or in patients who poorly tolerate pharmacological treatment.

## Author Contributions

The study was designed by RB, GS and CT. Patients selection and enrolment, collection of clinical and instrumental data was performed by EB, RB and CD in IRCCS Mondino Foundation, by AP in IRCCS Neuromed, by MR and GP in IRCCS Santa Maria Nascente, by MG and FM in Santa Lucia Foundation. Data was analyzed by EB and RDI and interpreted by RDI and ST. The manuscript was drafted by EB. AP, MR, RDI, CD, MG, ST, RB, GP, FM, GS and CT revised critically the manuscript. All authors approved the final version of the manuscript, and agreed to be accountable for all aspects of the work submitted.

## Conflict of Interest Statement

The authors declare that the research was conducted in the absence of any commercial or financial relationships that could be construed as a potential conflict of interest.
